# Optimizing human B cell repertoire analyses to interpret clinical data and design sequential HIV vaccines

**DOI:** 10.1038/s41541-025-01168-z

**Published:** 2025-07-17

**Authors:** Milton Maciel, Lindsey R. Baden, Brian Briney, Nicole A. Doria-Rose, Kasalina N. Kiwanuka, Shelly J. Krebs, Angela Malaspina, Georgia D. Tomaras, Andrew Ward, Gunilla B. Karlsson Hedestam, Wilton B. Williams, M. Patricia D’Souza

**Affiliations:** 1 Division of AIDS, National Institute of Allergy and Infectious Diseases, National Institutes of Health, Rockville, MD USA; 2 Brigham and Women’s Hospital and Harvard Medical School, Boston, MA USA; 3 Department of Immunology and Microbiology, The Scripps Research Institute, La Jolla, CA USA; 4 IAVI Neutralizing Antibody Center, The Scripps Research Institute, La Jolla, CA USA; 5 Consortium for HIV/AIDS Vaccine Development (CHAVD), The Scripps Research Institute, La Jolla, CA USA; 6 Department of Medicine, University of California at San Francisco, San Francisco, CA USA; 7 Vaccine Research Center, National Institute of Allergy and Infectious Diseases, National Institutes of Health, Bethesda, MD USA; 8 Military HIV Research Program, Walter Reed Army Institute of Research, Silver Spring, MD USA; 9 Center for Human Systems Immunology, Duke Human Vaccine Institute, Departments of Surgery and Integrative Immunobiology, Duke University, Durham, NC USA; 10 Department of Microbiology, Tumor and Cell Biology, Karolinska Institutet, 171 77 Stockholm, Sweden; 11 Duke Human Vaccine Institute, Departments of Surgery and Integrative Immunobiology, Duke School of Medicine, Durham, NC USA

**Keywords:** Immunology, Infectious diseases, Predictive markers

## Abstract

Advances in HIV vaccine development focus on eliciting broadly neutralizing antibodies (bNAbs) through next-generation immunogens. Human trials are testing these candidates for their ability to initiate B cell maturation. This workshop report reviews emerging data and methods for analyzing B cell repertoires, highlighting strategies to streamline these labor-intensive processes. The goal is to enable timely, cost-effective design of sequential immunization regimens that induce potent, protective bNAbs against HIV.

## Progress in engineering vaccine immunogens

Despite nearly four decades of scientific effort and investment, developing an HIV vaccine remains an elusive goal^1^. The challenges arise from the virus’s exceptional genetic diversity, its extensive immune evasion tactics, and its ability to quickly and permanently integrate into human immune cells^2^.

Early clinical trials of HIV vaccine candidates were primarily based on empirical approaches, examining various prime-boost combinations of HIV vaccine components expressed by viral vectors and/or the use of purified recombinant envelope (Env) glycoprotein preparations administered with adjuvants. However, none of the regimens tested so far have provided appreciable protection for clinical utility^3,4^.

Today, researchers are applying more systematic approaches to designing and developing vaccine immunogens aimed at eliciting cellular and humoral immunity against this highly variable virus. The goal is to eventually combine these approaches in a single vaccine regimen, as evidence suggests they may act synergistically^5^. The inclusion of Env-based immunogens that have the capacity to induce bNAbs is critical, as neutralizing antibodies are the correlate of protection for all known anti-viral vaccines^6^. Consequently, B cell-based vaccine candidates are more advanced, with several being studied in discovery medicine phase I clinical trials (DMCT), which are different from classical phase I trials in that they are designed for rapid and iterative assessment of vaccine strategies in humans to enable critical biologic insights to allow improved immunogen design^7^. A key objective of DMCTs is to rapidly characterize the vaccine-induced immune responses in depth from a given immunogen or sequence of immunogens to enable elicitation of improved immune responses, which are likely to be protective, such as bNAbs.

**Fig. 1:**
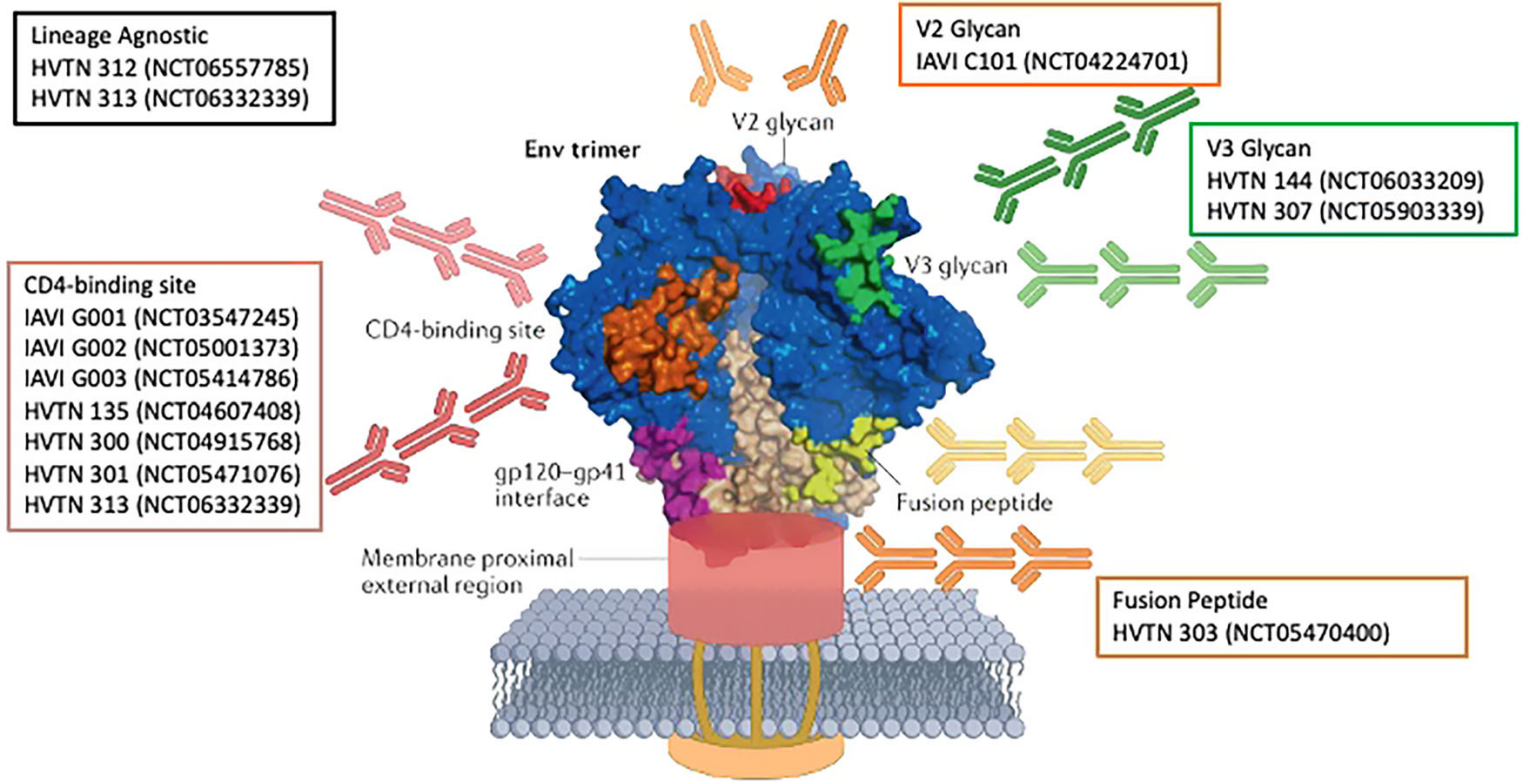
HIV Envelope protein and sites of vulnerability with respective broad neutralizing antibodies. Trial information eliciting epitope-specific antibodies shown. (Modified with permission from Haynes, B. F. et al. Strategies for HIV-1 vaccines that induce broadly neutralizing antibodies. *Nat. Rev. Immunol.* **23**, 142–158 (2023).

A globally effective HIV vaccine will likely need to elicit high and durable levels of bNAbs that protect against a significant portion of circulating viral variants. Results from the recently completed Antibody Mediated Prevention (AMP) trials support the use of Env immunogens that stimulate antibodies against determinants on Env that are functionally constrained and thus incompatible with immune escape mutations, referred to as sites of vulnerability, such as the CD4-binding site^8^. Importantly, several sites of vulnerability have been described for HIV Env (CD4-binding site, V2 apex, V3-glycan patch, fusion peptide, membrane proximal external region or MPER, and gp120-gp41 interface). Studies suggest that a broadly effective HIV vaccine should elicit antibodies targeting at least three of these epitopes^9^ (Fig. 1).

However, as research has shown, the induction of HIV-specific bNAbs is uniquely challenging. bNAbs exhibit several unusual characteristics that make them disfavored by the immune system^10,11^. In particular, bNAbs accrue many somatic hypermutations (SHMs) that account for their broad neutralization capability and potency, and some classes of bNAbs, such as those targeting the trimer V2 apex, have an unusually long heavy chain third complementarity-determining regions (HCDR3s)^9,12^. Studies show that naïve B cell lineages able to produce HIV bNAbs are relatively rare within the human B cell repertoire^13,14^. The rarity of bnAb precursor B cells, coupled with the fact that B cell lineage diversification through SHMs generates phylogenetic trees where only some branches may carry the mutations required for bNAb epitope binding, explains why bNAbs are observed in only a small fraction of people living with HIV (PLWH) and usually only appear after several years of infection. PLWH who make bNAbs typically only have one or a few B cell lineages that successfully mature into making bNAbs in response to ongoing virus evolution and B cell selection^9^.

Given these challenges, scientists have been compelled to design novel approaches for HIV vaccines. Several HIV vaccine candidates capable of initiating the complex process of B cell maturation required for bNAb development in appropriate animal models are currently undergoing evaluation in clinical trials. Rapid analysis and interpretation of immune responses in these studies are necessary to determine whether unique vaccine candidates can effectively elicit desired B cell responses. These analyses provide critical information for researchers in selecting optimal boosting immunogens intended to guide B cells toward the desired bNAb response. However, characterizing vaccine-induced HIV-specific repertoires at sufficient depth and across multiple vaccine recipients is labor-intensive. Therefore, new methods and bioinformatics pipelines are being developed to characterize the quality of B cell responses induced in vaccine trials at greater depth and in a cost-effective manner.

On 13 August 2024, experts in the HIV vaccine field gathered to discuss current analysis pipelines used to assess responses elicited by leading B cell-based HIV vaccine candidates at a virtual workshop sponsored by NIAID. Over 200 participants discussed ways to improve and harmonize the methodologies used to evaluate these candidate B cell-based HIV vaccines, including several next-generation sequencing methods. The workshop emphasized the importance of understanding how genetic variation in the human immunoglobulin (IG) loci influences B cell recognition of candidate immunogens and addressed the bottlenecks in characterizing human B cell repertoires. The primary focus of this workshop was to stimulate efforts to identify efficient and cost-effective methods for analyzing data from DMCT. This is crucial to expedite the development of an HIV vaccine regimen that can elicit potent bNAbs - a linchpin of an efficacious vaccine.

## Approaches to elicit HIV env-reactive bnAbs

Researchers are exploring several strategies to design vaccine immunogens that can induce antibodies against multiple neutralizing epitopes on the HIV Env glycoprotein. One strategy, referred to as germline targeting, involves using structure-based designs to reverse engineer an immunogen that can bind to and prime naïve B cells carrying B cell receptors (BCRs) with genetic properties that have the potential to develop into bNAbs against specific HIV Env epitopes. Additional immunogens developed for sequential administration should spur further SHMs in such bNAb precursor B cell lineages, guiding them to develop into bNAb-producing plasma cells.

A second strategy, referred to as mutation-guided B cell lineage approach, also aims to activate and expand rare naïve B cell clones. However, in this strategy, the maturation history of specific bNAbs isolated from PLWH is computationally reconstructed and employed to identify key improbable mutations that are required to achieve neutralization breadth. Subsequently, immunogens are developed to promote these mutations early in the B cell's response as a potential strategy to accelerate elicitation of vaccine-induced bNAb responses.

A third germline/lineage agnostic strategy focuses on engaging any naive B cell that recognizes bNAb target epitopes induced by native-like HIV Env trimers or epitope-based vaccines, which can be affinity matured through stepwise boosting using heterologous Env trimers to focus the response on conserved bNAb targets. Such approaches aim to drive the B cell response towards sites of vulnerability by exploiting the full potential of the highly polyclonal naïve B cell repertoire^15,16^.

All three strategies will require a series of immunizations with intervals that give enough time to promote affinity maturation of B cell lineages directed against bNAb targets. One of the most difficult obstacles in HIV vaccine development today is selecting the ideal immunogens to usher the maturation process and ultimately induce long-lived plasma cells capable of secreting bNAbs or long-lived memory B cells that can quickly respond to potential viral exposures following immunization.

## Evaluating B-cell vaccine immunogens

In recent years, researchers have made substantial progress in designing vaccine immunogens that prime naïve B cell precursors of HIV-specific bNAbs and initiate the stepwise process of maturing these responses.

Leo Stamatatos from the Fred Hutchinson Cancer Research Center shared data on the clinical testing of the germline targeting immunogen 426 c.Mod.Core nanoparticle, which was administered intramuscularly along with the adjuvants 3M-052-AF combined with aluminum hydroxide. This immunogen activates a range of B cell precursors of VRC01-class bNAbs, which target the critical CD4-binding site on HIV Env. In the HVTN 301 trial (NCT05471076), 48 volunteers from six sites across the U.S. received either full bolus or fractional doses^17^ of the 426 c.Mod.Core vaccine immunogen as a prime and full bolus as a boost. To date, 38 monoclonal antibodies induced by this vaccine have been isolated and characterized by biolayer interferometry (BLI), in vitro neutralization assays, and by Cryo-electron microscopy, which are revealing similarities in VRC-01 reactivity.

Bill Schief of The Scripps Research Institute, IAVI, and Moderna reported on a trio of trials (IAVI G001-NCT03547245, G002-NCT05001373, and G003-NCT05414786) involving another engineered germline targeting immunogen, the eOD-GT8 60-mer protein. This antigen is also designed to induce VRC01-class B cell precursors. In the G001 trial, the vaccine achieved a high response rate in 35 out of 36 participants (97% response rate)^18^. Only one individual in the trial did not generate detectable IgG B cells expressing VRC01 class B cell receptor precursors following two eOD-GT8 immunizations, since this individual lacked VRC01 permissive IGHV1-2 alleles (see section Understanding the role of genetic variation).

In the IAVI G002 and G003 trials, the eOD-GT8 60-mer immunogen was administered using Moderna’s mRNA platform. Initial observations indicate that the priming of VRC01-class B cell precursors was at least as effective with mRNA in the G002 vaccine recipients when compared to in the protein immunized G001 vaccine recipients. Standardized B-cell and serum antibody binding assays enabled direct comparison of the immunogenicity results of the two trials. Notably, the number of mutations in IGHV1-2-using VRC01-class monoclonal antibodies induced in G002 vaccine recipients was greater than in G001. These data support a role for mRNA vaccination to prime VRC01-class B cell precursors.

Rogier Sanders from Amsterdam University Medical Center presented data on another germline targeting B cell vaccine approach. This approach utilizes a native-like trimer immunogen, designated as BG505 SOSIP GT1.1, which is modified to bind to both VRC01-class and apex-specific B cell precursors. In infant macaques, after three immunizations, expanded VRC01-class B cells accumulated several mutations associated with VRC01-class bNAbs, suggesting that the antibodies elicited by BG505 SOSIP GT1.1 are substantially advanced on the path toward becoming bNAbs, according to Sanders^19^.

In addition to the efforts aimed at inducing VRC01-class (CD4-binding site) B cell precursors, other epitopes on the HIV envelope protein are being explored. Barton Haynes from Duke University, who leads the Consortium for HIV/AIDS Vaccine Development (CHAVD) funded by the NIAID, presented data from the HVTN 133 trial (NCT03934541), which tested an MPER peptide-liposome immunogen.

Although the HVTN 133 trial was stopped before the scheduled fourth and last immunization due to a case of anaphylaxis, Haynes shared insights from the analysis of B cell responses. The immunogen induced neutralizing-antibody responses capable of neutralizing some heterologous HIV strains. Although there were no substantial levels of circulating bnAbs, a monoclonal antibody isolated from a vaccinee neutralized 15% of tier-2 global HIV strains, and 35% of Clade B HIV strains, and represents an important benchmark for DMCT^20,21^. While a long interval is usually required for an HIV-1-induced bNAb B cell lineage to acquire the mutations required for breadth, in the HVTN 133, Haynes and colleagues demonstrated that MPER bNAb lineage was initiated after two immunizations in three out of five vaccinees, indicating affinity maturation to the target epitope.

These examples illustrate the importance of analyzing vaccine-induced antibody repertoires as deeply as possible. Participants of the workshop discussed the importance of sequencing as many immunogen-specific B cells as possible from each trial participant to assess the response following vaccination. Sequencing large numbers of antigen-specific paired heavy and light chains provides valuable information of antibody gene usage, clonality, and SHM. Schief noted that for germline-targeting immunogens, the effectiveness of the response can be directly evaluated from sequence information as the immunogens are intended to induce B cells with specific genetic features. Furthermore, sequencing longitudinal samples following priming and boosting provides information about the maturation of promising B cell lineages.

For the non-germline targeting approaches, sequencing alone does not provide sufficient information about epitope specificity and function. Efforts to measure serum neutralizing activity, antibody epitope mapping, including electron microscopy, polyclonal epitope mapping (EMPEM) studies^22^, and isolation and characterization of monoclonal antibodies (mAbs) are therefore needed. It was noted that these are critical complementary analyses also for germline-targeting approaches. A bottleneck in the evaluation of vaccine-induced responses is the ability to clone and express enough mAbs for functional and structural studies. All speakers emphasized the importance of thoroughly analyzing vaccine-induced antibody responses at depth, using both high-throughput sequencing and mAb isolation.

## Rapid and cost-effective evaluation of B cell immune responses

DMCTs are designed to answer specific questions about the immunogen, adjuvant, or immunization strategy as quickly as possible, thereby allowing researchers to rapidly and iteratively improve upon these results to expedite HIV vaccine development^7^. Weighing progress with bNAb-inducing vaccine immunogens depends on efficient, robust, and expeditious B cell sequencing, as well as functional and structural characterization of responses induced by each sequential immunization.

**Fig. 2:**
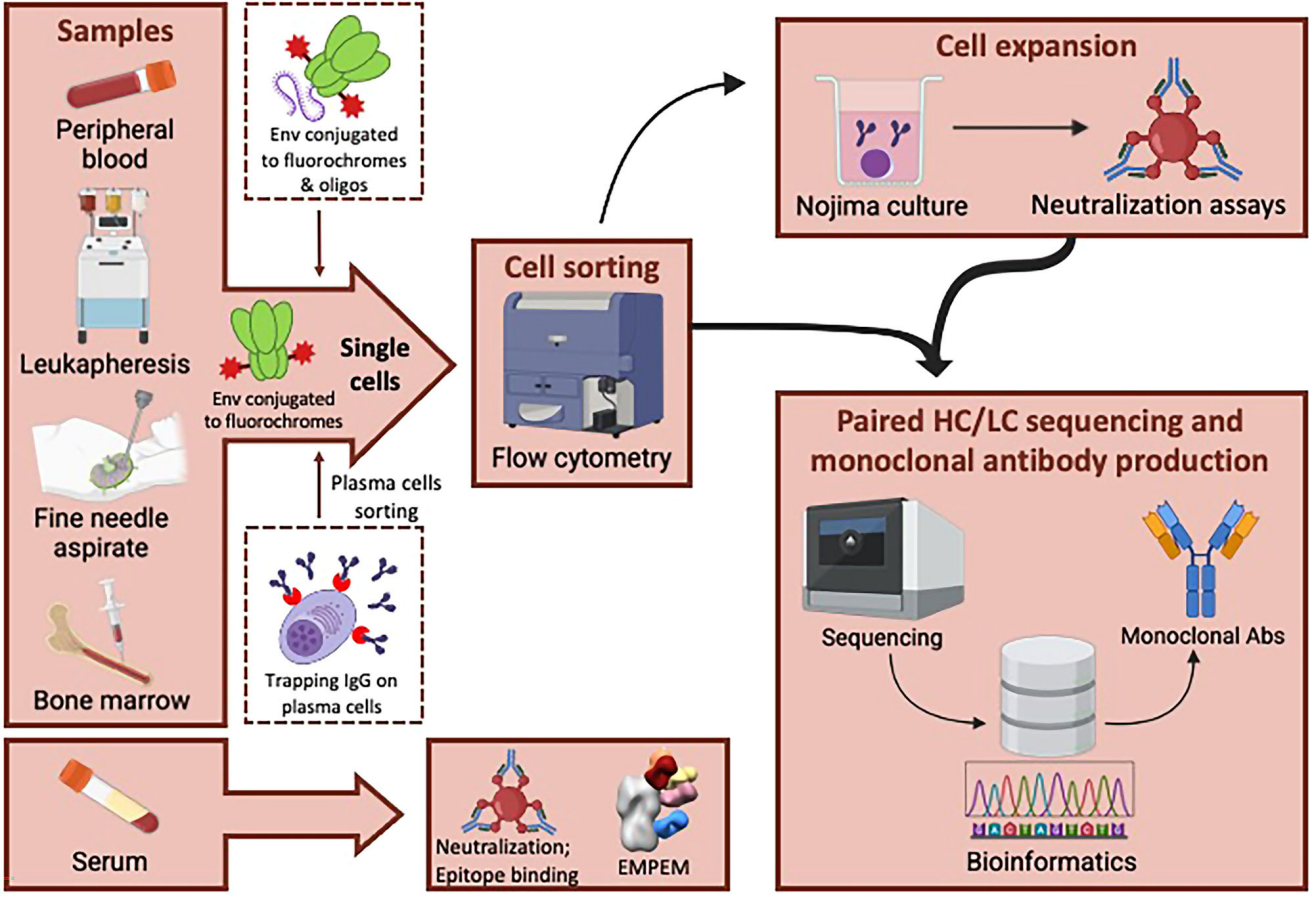
Sample collection and strategies for B cell sequencing and antibody response analysis. Samples from peripheral blood, leukapheresis, or fine needle aspirates (FNA) containing B cells can be sorted for isolation of single antigen-specific B cells by flow cytometry using antibodies against specific cell surface markers and fluorochrome- or oligo-conjugated Env proteins recognized by antigen-specific B cell receptors (BCRs). Bone marrow samples, containing plasma cells, can be sorted by using the TRAP assay. Nojima cultures from single B cells generate supernatants that can be screened for antibody binding and neutralization prior to sequencing. Antigen-specific B cells can be sequenced using different methodologies (10x Genomics, SMART-seq, etc), after which paired heavy and light chain HC/LC) V(D)J sequences are assigned to a relevant germline gene database and processed with different bioinformatics tools and pipelines. Serum samples can be analyzed for neutralizing activity, epitope-specific binding assays, and electron microscopy polyclonal antibody epitope mapping (EMPEM). Created with Biorender.com.

One critical factor in analyzing B cell-based vaccine regimens is sample collection. Researchers at the workshop reported the benefit of collecting larger volumes of peripheral blood from study volunteers—100 mL or more—along with leukapheresis, to maximize the number of B cells available for analysis. Additionally, fine needle aspirates (FNA) can be used to interrogate B- and T-follicular helper (Tfh) cell responses in lymph nodes, while bone marrow aspirates provide valuable insights into long-lived plasma cells through the antigen-specific mapping through immunoglobulin secretion, TRAP, and Sequencing (TRAPnSeq) methodology^23^ (Fig. 2).

Important considerations such as how many time points to analyze post-vaccination or whether it is more cost-effective and informative to conduct in depth analysis of the responses from a few individuals versus investigate a wider range of participants to better understand the overall vaccine response must be addressed in each trial, taking in consideration the primary goals, available resources, and budget.

Researchers employ a variety of approaches to sort and quantify epitope-specific, vaccine-induced B cell responses. Flow cytometry is a central methodology to isolate antigen-specific B-cells prior to sequencing. Rachel Parks from the HIV Vaccine Trial Network (HVTN) at Fred Hutchinson Cancer Center stressed that one of the biggest challenges in this process is selecting and developing high-quality probes to sort epitope-focused and antigen-specific B cells from various samples. Since the Env probes are study-specific and must be designed based on the immunogen used, access to expertise and facilities that can provide high-quality probes is an important component of vaccine evaluation. Such Env proteins are also needed to analyze antibody binding and for studies of mAbs complexed to the protein in structural studies.

After isolating the vaccine-specific B cells, a combination of methods for sequencing and functional evaluation of single B cells and the resulting mAbs are used to analyze and interpret the vaccine-induced responses (Fig. 2). These methodologies enhance the precision and sensitivity of the assays while improving overall throughput, thereby reducing analysis time and increasing cost effectiveness. During the workshop, speakers highlighted their experience with various techniques, including Nojima culture^24,25^, mRNA transcriptome analysis (e.g., 10X Genomics)^26^, SMART-seq (**S**witching **M**echanism **a**t the 5′ end of **R**NA **T**emplate **seq**uencing), RATP-Ig (**R**apid **A**ssembly, **T**ransfection and **P**roduction of **I**mmuno**g**lobulins)^27,28^, TRAPnSeq^23^, and the recently developed LIBRA-seq technology (**Li**nking **B cell R**eceptor to **A**ntigen Specificity through **Seq**uencing)^29^ (see Table 1).

**Table 1:** B-cell isolation and sequencing techniques

Technique	Pros	Cons
**Nojima culture** **Total time**: 33 days from sample thawing to harvesting supernatant for analysis. Time for further analysis (high throughput binding, TZM-bl assay, and sequencing) depends on the number of clones being analyzed. **Major bottleneck:** Time from sample to acquisition of supernatant for analysis is the longest compared to other techniques. refs. ^24,25^	• Easy setup for any institution with existing FACS-sorting and basic cell culture capabilities; • Eliminates in vitro heavy and light chain recombination; • Identifies antibodies produced by a single isolated B cell; • Cell pellets are compatible with most methods for BCR sequencing; • Can be paired with long-read sequencing systems suitable for long CDR3 clones (e.g., PacBio platforms Sequel II or Revio); • High recovery and data quality as millions of B cell receptors are sequenced from clones of a single FACS-isolated B cell.	• Terminally differentiated plasma cells cannot be isolated from samples using this method; • Cells must be viable and able to expand and differentiate in vitro; • Terminally differentiated B cells cannot be attained in vitro for study and/or analysis.
**10x genomics™ platform** High-throughput single-cell transcriptomes, barcoded antigen signals, and immune repertoire (VDJ) sequencing. **Total time**: ~14 days from sample thawing to generation of sequencing data. **Major bottleneck:** Trial-specific reagents increase the specificity of this method; however, this can create a bottleneck due to the associated manufacturing and production costs. 10x Genomics requires a high number of cells (When possible, a minimum of ~100,000-150,000 cells, with a concentration of ~1000-1600 cells per microliter) and ~90% cell viability for successful sequence recovery, necessitating the use of various approaches to reach this threshold. Therefore, it is critical to optimize this method. ref. ^26^	• Allows isolation and sequencing of B cell receptors with multiple epitope specificities; • Quantification of vaccine-specific memory B cells by flow cytometry is obtained while sorting cells for genomic analysis; • CITE-Seq^a^ antibodies uniquely label each sample, facilitating the identification of individual samples after sequencing; • Platform permits the analysis of multiple aspects of the isolated cells: B cell receptors, cell surface proteins, and gene expression; • It can be paired with a systems the NovaSeq 6000™, that is capable of large-scale whole-genome sequencing, suitable sequencing large number of genomes/run; • In-house bioinformatic pipeline allows for study-specific data processing.	• Fluorescent probes specific to the study are required to characterize and isolate vaccine-specific B cell clones by flow cytometry; • Probe design must consider the study objectives (e.g., knock-out versions); • Probes must be validated through the production of monoclonal antibodies and measurement of biomolecular interactions.
**RATP-Ig** (Rapid assembly, transfection, and production of immunoglobulins) **Total time**: ~7 days from cell isolation to antibody cultures and functional characterization. **Major bottleneck:** When paired with Illumina SMART-Seq platform, which is very helpful for analysis of limited sample volume, like single cells or very small RNA quantities, the throughput is significantly limited. When paired with 10x Genomics™ functional analyses are no longer be possible. refs. ^27,28^	• SMART-Seq^b^ requires sorting single cells into individual wells, which helps preserve the unique features of each isolated cell during subsequent processing and analysis (e.g., VDJ pairing, gene transcription, and flow cytometry profiling); • Addition of non-templated C residues at the 5’ end of the generated cDNA molecule provides an anchor for specific TS oligomers. This modification ensures better coverage of the entire mRNA molecule and allows for analysis of only fully synthesized cDNA. This also improves the enrichment of amplified Ig sequences. • Enable rapid acquisition of functional antibodies from individual clones.	• Sorting one cell per well per plate for SMART-Seq allows only 96 cells per plate; • Laborious process as VDJ cassettes must be generated, inserted into the enriched immunoglobulin amplicons, transfected into mammalian cells, and finally, the transformed cells must be cultured to obtain antibody containing supernatants.
**LIBRA-seq** (Linking B cell Receptor to Antigen Specificity through Sequencing) **Total time**: ~2–6 weeks from sample thaw and cell isolation to sequencing. **Major bottleneck:** Sequencing performed with 10x Genomics™ platforms to recover DNA-barcoded antigens and *B cell* receptor sequences simultaneously (10x Genomics™ limitation described above). ref. ^29^	• Multiple methods to fluorescently label the antigen of interest (direct vs indirect) • Allows screening of multiple antigens simultaneously; • Allows analysis of larger matrix analysis of B cell specificities; • Straightforward and compatible with antibody synthesis; • Easily modified to include “knock out” proteins for epitope screening (see 10x Genomics platform above); • Easily adaptable for other species, e.g., guinea pig.	• Sensitivity needs improvement; LIBRA-seq method does not always identify the specific antigen for all tested clones (ongoing optimization of antigen barcoding).
**TRAPnSeq assay** (Antigen Specificity Mapping through Immunoglobulin [Ig] Secretion TRAP and Sequencing) This assay may be paired with either SMART-Seq or 10x Genomics™ platforms. **Total time**: ~14 days from sample thawing to generation of sequencing data. **Major bottleneck:** Similar to RATP-Ig, when paired with SMART-Seq for low sample volume, the throughput is significantly limited. When paired with 10x Genomics™, functional analyses are no longer possible. ref. ^23^	• Allows antibody isolation of plasma cells; • When paired with 10x Genomics allows for the integration of cells from multiple samples into a single experiment; • When paired with SMART-Seq, it preserves the unique features of the isolated cell and ensures better coverage of the entire mRNA molecule (see SMART-Seq technique above).	• Samples processed for use with 10x Genomics™ platform may lose the unique features of individual cells (e.g., VDJ pairing, gene transcription, flow cytometry profile). This issue may be overcome by using oligo-markers instead of fluorescent-tagged markers. • With SMART-Seq, sorting one cell per well on a plate allows for the processing of only 96 cells per plate.

^a^Cellular indexing of transcriptomes and epitopes by sequencing (simultaneously quantifies transcription and protein expression).

^b^Switching mechanism at 5' end of RNA template.

Each of these strategies offers advantages and disadvantages in terms of timing, output, sensitivity, and cost. Thus, there should be careful consideration to appropriately select the assay of choice based on the scientific question asked for immune monitoring of clinical trials. Experts at the workshop suggested that a concentrated effort to increase sample input while decreasing the costs of these labor-intensive analyses will help expedite the evaluation of samples in DMCT. This effort will likely require the advancement of automation for B-cell sequencing and close collaboration between wet-lab and computational scientists. Regardless of the methodology employed, the quality of the antibody sequence data depends on the integration of state-of-the-art bioinformatics, statistical, and data science tools to analyze vaccine-induced responses at high resolution.

Critical to all HIV vaccine trials is the evaluation of neutralizing activity of polyclonal antibodies in serum, since the elicitation of such responses is the ultimate objective for a protective vaccine. The TZM-bl pseudovirus neutralization assay is most frequently used^30,31^. Researchers at the workshop emphasized the importance of having well-characterized virus panels representing global HIV diversity, including tier 2 viruses from all clades and relevant negative controls. Neutralizing activity is an important measure to follow the evolution of vaccine-induced responses in DMCTs. Current bNAb precursor-inducing vaccines, including germline-targeting vaccines, are not expected to generate neutralizing antibodies after priming alone, thus, the development of precursor-sensitive HIV strains described by David Montefiore from Duke University is another important tool in evaluating bNAb-inducing responses. While autologous neutralization is achieved in some trials, the generation of mAbs with neutralization breadth for clinical utility is expected to take time and multiple boosts, and as of yet, this has not been consistently achieved.

## Understanding the role of genetic variation

The recognition of antigens by BCRs is the first step in B lymphocyte activation. While BCRs are encoded within genetic loci that are known to be highly diverse^32,33^, the extent of this variation and functional impact on the human antibody response remain largely unknown. Knowledge about allelic variation in the genes encoding BCRs is critically important for understanding adaptive immune responses and identifying responder and non-responder phenotypes for vaccination strategies that aim to engage specific B cell lineages to induce HIV-specific bNAbs. For instance, in the IAVI G001 trial, there was a single non-responder of 36 participants. Genotyping of the participants’ immunoglobulin heavy chain variable (IGHV) genes using the IgDiscover genotyping tool developed in the Karlsson Hedestam laboratory at the Karolinska Institutet^34^ showed that this non-responder lacked the IGHV1-2 alleles (*02 and *04) required to generate VRC01-class responses^18,35^. These results demonstrated the need to understand population-level Ig allelic variations when designing germline-targeting immunogens aimed for clinical evaluation. Further analysis of the G001 trial revealed that genotypic variation was a greater predictor of vaccine-induced VRC01 class bNAb B cell response among vaccine responders than vaccine dose, since IGHV1-2*02 is more frequently used in B cell repertoires than IGHV1-2*04. In fact, IGHV1-2*02 homozygous persons responded better than IGHV1-2*02/*04 heterozygous persons or IGHV1-2*04 homozygous persons, as shown by deCamp and colleagues^35^. An estimated 2% of individuals in the IAVI G001 study population, conducted at sites in North America, lack at least one of the alleles required to induce VRC01-class precursor B cells, underscoring the importance of performing genotypic analyses when interpreting the results of germline-targeting HIV vaccine trials.

Unlike human leukocyte antigen (HLA) loci, which are well-characterized at the population level, the Ig loci have not been extensively studied on a large scale. Current reference databases cataloging allelic variants of Ig genes are, therefore, inadequate. The International Immunogenetics Information System (IMGT) database was established in 1989 as a repository for Ig and T-cell receptor germline sequences. However, almost half of the alleles in the IMGT database have not been independently confirmed, and the alleles were primarily identified in studies involving individuals of European descent.

To address these shortcomings, Martin Corcoran of Karolinska Institutet described ongoing work to develop improved databases. He began by mentioning that the Adaptive Immune Receptor Repertoire (AIRR) community of The Antibody Society recently published a database, the AIRR-C reference set, which was curated to only include alleles from IMGT that have been independently validated^36^. In addition, he described ongoing work in the Karlsson Hedestam laboratory using data from ~2500 persons from the 1000 Genomes Project^37^, which they have used to define Ig variation in populations worldwide. Through this effort, they identified the greatest Ig allelic variation in sub-Saharan African populations with multiple novel alleles for each IGHV gene. Overall, 25 population groups were studied, and frequencies of each allele in different parts of the world were defined, revealing population diversity of great relevance for HIV vaccine development. Based on this information, the workshop participants discussed the importance of having informed consent to genotype participants in vaccine trials, even for whole genome sequencing as is currently being done at the Vaccine Research Center.

Researchers at the workshop emphasized that understanding and characterizing the enormous population-level diversity of the Ig loci on a global scale is a priority for the field. This information will play a critical role in designing clinical trials of germline-targeting HIV vaccine strategies and analyses of the results. As these data are collected, researchers aim to associate specific genotypes with the functionality of antibody responses.

## Addressing bottlenecks to expedite future research

The challenges associated with developing an effective HIV vaccine have led new approaches to vaccine design, including testing germline-targeting, mutation-guided, and the use of natively folded Env trimers or epitope-based immunogens in DMCT. The success of these trials depends on the efficient and carefully executed genetic, functional, and structural analysis of the B cell and antibody responses induced in these clinical studies.

These analyses include sophisticated B cell receptor sequencing of large number of antigen-specific Ig genes, isolation of mAbs, and structural evaluation. When coupled with comprehensive serological studies of polyclonal responses, these detailed workflows can indicate whether the vaccine-induced immune responses are on the path towards HIV-specific bNAb development and, importantly, guide the design of future immunogens in a sequential immunization strategy.

As more vaccine immunogens and combination approaches are tested in clinical trials, it will become critical to optimize the analysis and bioinformatics pipelines to increase efficiency and lower cost, while maximizing the data collected for each strategy. This workshop highlighted bottlenecks in these pipelines and helped identify future priorities for the field (see Table 2). Addressing these issues will expedite the development of bNAb-based HIV vaccine candidates to be effective on a global scale.

**Table 2:** Recommendations and priorities for the field

**• Enhance antibody cloning -** Implement higher throughput antibody cloning methods to sample a broader range of the vaccine-induced B cell repertoire, thereby improving cost effectiveness.
**• Further automate sequencing steps:** Streamline B cell-sequencing process through automation to enable rapid evaluation of various aspects of the vaccine-induced response.
**• Promote collaboration:** Foster close collaboration between wet-lab scientists and computational experts to continuously optimize analytical and bioinformatics workflows.
• **Address population diversity:** Expand genotypic testing of Ig germline genes on a global scale.
• **Use Ig allelic information:** Leverage Ig allelic data to select vaccine candidates to achieve broad coverage outcomes of clinical studies.

## Data Availability

No datasets were generated or analysed during the current study.
